# Recent Advances in IRAK1: Pharmacological and Therapeutic Aspects

**DOI:** 10.3390/molecules29102226

**Published:** 2024-05-09

**Authors:** Kyeong Min Kim, Na-Hee Hwang, Ja-Shil Hyun, Dongyun Shin

**Affiliations:** College of Pharmacy, Gachon University, Hambakmoe-ro 191, Yeunsu-gu, Incheon 21935, Republic of Korea

**Keywords:** interleukin receptor-associated kinase (IRAK) proteins, inhibitor, degrader, immunity

## Abstract

Interleukin receptor-associated kinase (IRAK) proteins are pivotal in interleukin-1 and Toll-like receptor-mediated signaling pathways. They play essential roles in innate immunity and inflammation. This review analyzes and discusses the physiological functions of IRAK1 and its associated diseases. IRAK1 is involved in a wide range of diseases such as dry eye, which highlights its potential as a therapeutic target under various conditions. Various IRAK1 inhibitors, including Pacritinib and Rosoxacin, show therapeutic potential against malignancies and inflammatory diseases. The covalent IRAK1 inhibitor JH-X-119-01 shows promise in B-cell lymphomas, emphasizing the significance of covalent bonds in its activity. Additionally, the emergence of selective IRAK1 degraders, such as JNJ-101, provides a novel strategy by targeting the scaffolding function of IRAK1. Thus, the evolving landscape of IRAK1-targeted approaches provides promising avenues for increasingly safe and effective therapeutic interventions for various diseases.

## 1. Introduction

Interleukin receptor-associated kinase (IRAK) proteins are a class of serine/threonine kinases that act as primary regulators of interleukin (IL)-1R signaling and Toll-like receptor (TLR)-mediated signaling. These pathways are crucial for regulating innate immunity and inflammation [1,2]. These pathways are triggered by various IL-1 family cytokines, protein-associated molecular patterns (PAMPs), and damage-associated molecular patterns (DAMPs) that activate TLR or IL-1R [3]. Upon activation, the intracellular domains of Toll/interleukin-1 receptors within TLR dimers trigger the formation of a multiprotein signaling complex known as the myddosome, which includes MyD88 and members of the IRAK family [4,5].

**Figure 1:**
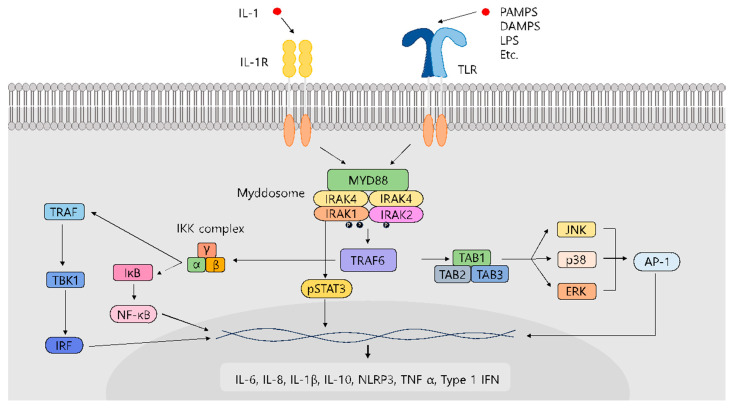
Pathway of interleukin-1 signaling (IL-1R) and Toll-like receptor (TLR)-mediated signaling.

Initially, MyD88 is recruited by most TLRs, except for TLR3 or IL-1R, leading to the subsequent recruitment of IRAK members [6]. Following the binding of the ligand to IL-1R or TLRs, IRAK undergoes phosphorylation, leading to its dissociation from the receptor complex and its association with tumor necrosis factor (TNF) receptor-activated factor 6 (TRAF6) [7]. Subsequently, TRAF6 initiates signaling cascades downstream, leading to the activation of NF-κB and various stress kinases, including c-Jun NH_2_-terminal kinase (JNK) and p38 mitogen-activated protein kinase (MAPK) [8]. As these pathways play a role in various pathophysiological conditions, IRAKs are crucial contributors to the pathophysiology of cancers and metabolic and inflammatory diseases [9,10,11]. Therefore, IRAK mutations are frequently linked to increased susceptibility to various diseases, including inflammatory diseases, viral diseases, and cancer [11,12] (Figure 1). Thus, inhibiting IRAKs could exert potential therapeutic benefits.

The IRAK family comprises four mammalian members: IRAK1, IRAK2, IRAK3 (IRAK M), and IRAK4. Despite all IRAKs being classified as serine/threonine protein kinases and sharing a kinase-like domain, only IRAK1 and IRAK4 exhibit kinase activity. IRAK3 and possibly IRAK2 are pseudokinases without active catalytic functions; however, they may still play important roles in signaling pathways [13]. IRAK2 is a fundamental component of the IL-IR signaling complex like IRAK1 [14], and IRAK3 acts as a negative regulator of the TLR pathway and may therefore reduce the innate immune response by inhibiting the dissociation of IRAK1 or IRAK2 from myddosome complexes [15]. IRAK4 is central to IL-1R/TLR signaling. Moreover, as an upstream activator of IRAK1, it phosphorylates IRAK1 [16]. IRAK1 was the first member of the IRAK family to be discovered and has diverse roles in controlling inflammation [17]. IRAK1 functions as a key link in TLR/IL-1R signaling in addition to its involvement in interferon regulatory factor (IRF) activation and rapid NLRP3 (nucleotide-binding domain, leucine-rich-containing family, and pyrin domain-containing-3) inflammasome activation [18,19]. TLR signaling and pro-inflammatory cytokine production in human macrophages primarily depend on IRAK1 and are less affected by disturbances in IRAK4 or IRAK2 [20]. Moreover, IRAK1 plays a crucial role in various diseases [21], and several preclinical studies indicate potential clinical benefits in the selective inhibition of IRAK1 [22,23,24].

Therefore, IRAK1 is a potential disease-modifying target; various modalities of modulators are being explored to regulate it. In this review, we focus on IRAK1 and discuss its physiological functions and related diseases. Furthermore, we present an overview of clinical trials involving various modulators, including IRAK1/4 dual inhibitors, selective IRAK1 inhibitors, selective covalent inhibitors, and selective IRAK1 degraders. Particularly, this review addresses the advantages of selective IRAK1 inhibitors or degraders over dual IRAK1/4 or selective IRAK4 inhibitors.

## 2. IRAK1-Related Disease

### 2.1. Immune Disease

The innate immune system rapidly responds to infections through sensitive and efficient mechanisms. The TLR and IL-1R families detect pathogens in response to inflammatory cytokines. IRAKs are involved in these processes and play a crucial role in promoting the expression of inflammatory genes in these signaling pathways [17].

#### 2.1.1. Inflammatory Disease

IRAK1 is an essential component of the IL-1 signaling pathway that has been implicated in various inflammatory diseases.

##### Sepsis

Sepsis is a severe condition where the response of the body to an infection triggers widespread inflammation, leading to organ dysfunction. Sepsis may be life-threatening and requires prompt medical attention. The major factors contributing to sepsis are abnormal inflammation and inadequate oxygen supply to tissues and muscles [25]. In 2013, Chandra et al. [26] demonstrated that IRAK1 deficiency affects multiple TLR-dependent pathways and diminishes the early cytokine response following polymicrobial sepsis. The delayed onset of inflammation due to IRAK1 deficiency indicates its crucial role in early postseptic signaling via TLR4 and TLR7/8. In contrast, the inflammatory response during the later stages of sepsis appears to be triggered by alternative TLR-dependent pathways or overlapping signaling mechanisms [26]. MicroRNA-146a (miR-146a) was first found by Taganov et al. in 2006 who revealed its role as a suppressor in the innate immune system and in mitigating inflammatory reactions triggered by LPS [27,28]. Some notable results were published that miR-146a downregulates IRAK1 and other cytokines in LPS-induced immune, inflammation, or sepsis responses, demonstrating the direct relationship between miR-146a and IRAK1. Gao et al. revealed that transfecting with miR-146a attenuated NF-kB activity induced by sepsis, suppressed IRAK1 and TRAF6 expression in the heart muscle, and reduced the production of inflammatory cytokines induced by sepsis. Their result suggested that miR-146a might have a protective effect on sepsis-induced cardiac dysfunction by targeting IRAK and TRAF6 [29].

##### Fibrotic Disease—Myelofibrosis (MF)

Myelofibrosis (MF) is a chronic bone marrow disorder characterized by excessive scar tissue formation and the disruption of normal blood cell production. MF may lead to anemia, fatigue, an enlarged spleen, and other complications. MF presents two phenotypes: cytopenic and myeloproliferative. Cytopenic myelofibrosis can be potentially cured by the deregulation of the immune state based on the myddosome–IRAK–NFκB axis [30]. The engagement of TIR (Toll/interleukin-1R) domain receptors leads to dimerization and myddosome formation by recruiting the adaptor protein MyD88. The MyD88 N-terminal death domains interact with IRAK family kinases to form active signaling complexes. Following IRAK1 trans-phosphorylation, this complex activates TRAF6, leading to the induction of inflammatory cytokines and interferons. Additionally, miR-146a downregulates myddosome signaling by targeting IRAK-1, TRAF-6, and TGF-β transcripts [27]. It ultimately increases inflammatory cytokines and inhibits IRAK1, leading to the suppression of these cytokines and a decrease in CD34 + colony formation, but not of JAK1 or JAK2 [31,32].

##### GVHD

Graft-versus-host disease (GVHD) is a complication of stem cell or bone marrow transplantation. This occurs when the transplanted cells attack the recipient tissues, leading to various health issues. IRAK1 controls the activation of antigen-presenting cells (APCs) under inflammatory conditions, and suppressing IRAK1 may potentially reduce APC activation and alleviate acute graft-versus-host disease (aGVHD) [33]. IRAK1 inhibition indicates its potential to impede T-cell differentiation. IL-12 and IFNγ derived from APCs instruct effector T-cells to differentiate into IFNγ-producing helper T-cells or cytotoxic T-cells [34]. The newly identified compound Jh-X-119-01 exhibits a highly selective inhibitory effect on IRAK1 [35].

##### Dry Eye

Inflammation is the primary cause of dry eye disease (DED). The overexpression of miR-146a reduces the expression of its targets, IRAK1 and TRAF6, and inhibits the nuclear translocation of NF-κB p65 from the cytoplasm. Moreover, miR-146a mitigates TNF-α-induced expression of IL-6, IL-8, COX2, and intercellular adhesion molecule 1 (ICAM1), suggesting that miR-146a-mediated inhibition of IRAK1 could potentially mediate the inflammatory response in DED [36]. Additionally, Yin et al. [37] have shown that miRNA-146a-5p suppresses IRAK1, IL-6, TNF-α, and CBP expression, which reduces the inflammatory response in DED.

#### 2.1.2. Autoimmune Disease

##### SLE

Systemic lupus erythematosus (SLE) is an autoimmune disease that affects multiple organs, causing joint pain, skin rashes, and fatigue. Genomic study results obtained by Jacob et al. [38] have shown an association between IRAK1 and vulnerability to SLE. Notably, IRAK1 expression in CD4+ T-cells is increased in individuals with SLE. IRAK1 overexpression is observed even in patients with SLE with inactive disease, indicating that it may be inherently and consistently increased in SLE, irrespective of the disease activity level [39].

##### Autoimmune Hypophysitis

Autoimmune hypophysitis is the inflammation of the pituitary gland caused by the host immune system attacking its own cells. This can disrupt hormone regulation, leading to various symptoms and hormonal imbalances. It is a rare condition that is diagnosed through imaging and hormone-level assessments and may be managed with hormone replacement therapy or immunosuppressive medications in severe cases. IRAK1 levels were elevated in the pituitary glands of mice that developed experimental autoimmune hypophysitis (EAH). The use of an IRAK1-specific inhibitor, rosoxacin, may suppress EAH by hindering the activation and/or differentiation of autoreactive T-cells in pituitary-draining lymph nodes or by preventing T-cell infiltration into the pituitary gland [40].

#### 2.1.3. Cardiovascular Disease

Cardiovascular disease (CVD) encompasses various conditions that affect the heart and blood vessels, with potentially serious outcomes such as heart attack and stroke [21]. Both innate and adaptive immune systems are crucial for maintaining balance and influencing CVD development [39]. The release of DAMPS during I/R injury (ischemia–reperfusion injury) and endothelial cell injury may also activate the inflammatory system [41].

##### Atherosclerosis

Atherosclerosis, which is commonly referred to as arteriosclerosis, is a chronic inflammatory condition characterized by the buildup of lipid-laden foam cells in the arterial wall [42]. ABCA1 is essential for reverse cholesterol transport, as it removes excess cholesterol from peripheral tissues, like macrophages in artery walls, and transports it to the liver for disposal. The impact of TLR4 signaling on ABCA1 (ATP-binding cassette transporter A1) expression and lipid accumulation is linked with IRAK1 involvement. Silencing IRAK1 expression by using a targeted siRNA reversed the TLR4-induced downregulation of ABCA1 and subsequent lipid accumulation in vitro and in vivo [43].

#### 2.1.4. Neurodegenerative Diseases

Neuroinflammation is believed to play a significant role in the development and progression of most of neurodegenerative diseases such as Alzheimer’s disease and Parkinson’s disease [44,45].

##### Alzheimer’s Disease (AD)

Alzheimer’s disease is a representative chronic neurodegenerative disorder characterized by memory loss, cognitive decline, and changes in behavior. It is the most common cause of dementia, particularly in elderly individuals. Historically, the accumulation of amyloid plaques and/or neurofibrillary tangles is understood to be a leading cause of the disease. Recently, increasing evidence proves that neuroinflammation of astrocytes and microglia can promote AD [46,47,48]. In the neuroinflammatory process, IRAK4 and IRAK1, as downstream signaling components of TLRs and IL-1s, are crucial and the inhibition of IRAK1/4 was revealed to reduce neuroinflammation and present potential as a new target for AD [49,50].

### 2.2. Oncology

In 1863, Rudolf Virchow reported the association between inflammation and cancer after discovering the infiltration of white blood cells into tumor tissues. TLRs play a role in the response of numerous cancer cells. Hence, the IRAK family of kinases, which are essential mediators in IL-1R signaling pathways and TLR inflammation, could be rational drug candidates for diverse malignancies [11]. Additionally, IRAK1 expression is elevated in nearly every tumor type, with the exception of thyroid carcinoma (THCA) [51].

#### 2.2.1. Solid Tumor Malignancies

In 2004, Pilarsky et al. reported that IRAK1 is a significant marker of solid tumors. Recent studies have suggested that targeting IRAK1 is an effective treatment for diverse solid tumors [52].

##### Glioma

Glioma is a type of brain tumor that originates from glial and supportive cells of the central nervous system. Satisfactory treatments for gliomas are currently lacking, and the average survival for patients with glioblastoma (GBM) is <15 months, underscoring the need for new therapeutic approaches [53]. IRAK1 expression is significantly upregulated in neuroblastoma tissues compared with that in benign brain tumor tissues. This indicates poor prognosis [54]. A key discovery of that previous study was the upregulation of IRAK1 expression in response to ionizing radiation (IR) treatment. IR-induced DNA damage leads to cytosolic dsDNA accumulation in tumor cells, activating the cGAS (cyclic GMP-AMP synthase)-STING (stimulator of interferon genes) pathway. This pathway involves cGAS, which detects dsDNA, initiates cGAMP (cyclic GMP-AMP) synthesis, and activates STING. Activated STING, upon translocation to the Golgi complex, triggers TANK-binding kinase 1, which activates IRF3 and NF-κB and ultimately induces type-I IFN. As IRAK1 is an upstream component in the NF-κB pathway and crucial for IFN-I production, a previous study explores the potential mediation of IR-induced IRAK1 expression via the STING pathway [55,56].

##### Breast Cancer

Triple-negative breast cancer (TNBC) is a subtype characterized by the absence of estrogen and progesterone receptors and HER2. It is known for its aggressiveness, and its treatment typically involves chemotherapy because hormone- and HER2-targeted therapies are ineffective. In 2015, Wee et al. [57] reported that IRAK1 is notably overexpressed in TNBC, and its overexpression is associated with conferring growth advantages to TNBC through the secretion of NF-κB-related cytokines. Significant upregulation of miR-146a-5p effectively inhibited the proliferation, invasion, and migration of breast cancer cells (MDA-MB-453 and MCF7). Additionally, miR-146a-5p directly targeted the 3′-untranslated IRAK1 region, leading to its downregulation in breast cancer cells [58]. Li et al. [59] showed that miR-146a increased the response of breast cancer cells to PTX (paclitaxel) by reducing IRAK1 expression.

##### Endometrial Cancer

Tumor-associated macrophages (TAMs) promote epithelial–mesenchymal transition (EMT) in endometrial cancer (EC) cells and suppress apoptosis. Elevated Arg-1 (arginase-1) expression, which is a marker of M2-polarized macrophages that secrete anti-inflammatory cytokines such as IL-10 and IL-4, is observed in TAMs. IRAK1 is a downstream target of miR-192-5p. Increased miR-192-5p levels in TAM-derived exosomes suppress IRAK1 expression in EC cells, leading to the deactivation of NF-κB signaling. In vivo, miR-192-5p overexpression in TAM-derived exosomes inhibit IRAK1 and NF-κB expression in tumor tissues and downregulated NF-κB phosphorylation [60].

##### Hepatocellular Carcinoma

Hepatocellular carcinoma (HCC) is the most common form of primary liver cancer that originates in hepatocytes. It is often linked to chronic liver diseases like cirrhosis, with risk factors including viral hepatitis and alcohol-related liver disease; hence, inflammation is deeply related to HCC. The overexpression of SMARCA4, SMARCC1, and SMARCA2 genes, which belong to the SWI/SNF subunit, has been confirmed in human HCC. Specifically, SMARCA4 has been proven to activate IRAK1 expression in HCC through the IRAK1 enhancer. The transcriptional activation of IRAK1 in HCC leads to the induction of the tumor proteins gankyrin and aldo-keto reductase family 1 member B10 (AKR1B10) [61]. According to Chen et al. [62], suppressing IRAK1 through knockdown impedes HCC progression by preventing NLRP3 from obstructing the MAPKs/IL-1β pathway. These findings suggest a potential strategy for treating HCC.

##### Prostate Cancer

Prostate cancer (PCa) has been ranked as the second most common malignancy (following lung cancer) [63]. According to Oseni et al. [64], IRAK1 is typically upregulated at both the mRNA and protein levels, and genetic mutations have been observed in clinical PCa samples. Moreover, IRAK1 deregulation not only reduces cell viability, cellular migration, and inflammatory cytokine production but also increases cell death in a dose- and time-dependent manner.

Notably, IRAK1 is concentrated in luminal epithelial cell clusters, whereas IRAK4 and MYD88 are predominantly expressed in the leukocyte cluster, and TRAF6 is observed in the endothelial cell cluster [65]. Additionally, a previous study showed that the functional role of IRAK1 does not rely strictly on its catalytic activity, as catalytically inactive forms of IRAK1 that are marked by a point mutation at the catalytic site or a splice variant missing a portion of the kinase domain trigger NF-kB activation [66,67]. This has obvious implications for potential therapeutics inhibiting kinase activity.

#### 2.2.2. Hematologic Malignancy

##### Mutated B-Cell Lymphoma—ABC-DLBCL

Activated B-cell-type diffuse large B-cell lymphoma (ABC-DLBCL) is a subtype of diffuse large B-cell lymphoma (DLBCL), which is a type of non-Hodgkin lymphoma. These lymphomas are often aggressive and may have distinct clinical and biological characteristics compared with those of other DLBCL subtypes.

IRAK1 genes play a crucial role in ABC-DLBCL with MyD88 mutations [68]. Survival of ABC-DLBCL tumors predominantly relies on ongoing nuclear NF-κB signaling, driven by genetic alterations that activate the B-cell receptor (BCR) and TLR pathways [69]. Sustained MyD88-IRAK signaling is essential for both ABC-DLBCL progression and tumor cell viability [11]. Significantly, the scaffolding function of IRAK1 and not its kinase activity is essential for tumor cell survival in this context [68]. This has obvious implications for potential therapeutics inhibiting kinase activity.

##### AML

Acute myeloid leukemia (AML) is a cancer that affects the blood and bone marrow—specifically the myeloid cells. In AML, immature white blood cells (myeloblasts) are overproduced, which interferes with the normal functioning of the bone marrow. AML exhibits heightened IRAK1 expression, offering a survival signal to AML cells. Genetic suppression of IRAK1 in primary AML samples and xenograft models results in a substantial decrease in leukemia burden [70].

### 2.3. Viral Infection

Recent studies have highlighted the pivotal role of the IRAK family in the TLR and IL-1 receptor signaling pathways and established its association with infections such as SARS-CoV-2 (COVID-19) and human immunodeficiency virus type 1 (HIV-1) [71].

#### HIV/SARS-CoV-2

The progression of HIV-1 is linked to ongoing immune activation and persistent inflammation even in the presence of successful viral suppression through antiretroviral treatment (ART) [72]. Chronic inflammation and hyperimmune activation persist in individuals receiving ART for HIV-1 infection. Elevated circulating levels of inflammatory biomarkers such as IL-1β, IL-6, and TNF are indicative of the risk of comorbidities and mortality [73,74]. A small fraction of COVID-19 patients with SARS-CoV-2 undergo cytokine release syndrome (CRS), which is commonly referred to as a cytokine storm. This underscores the potential dangers of widespread immune-mediated lung impairment associated with these diseases [75,76,77,78,79].

NLRP3 has been implicated in the pathogenesis of both diseases. IRAK1 is a crucial component of TLR-mediated NLRP3 inflammasome activation and MyD88 regulation and exerts a potential therapeutic effect by modulating multiple IL-1- and TLR-mediated signaling pathways that regulate immune response and inflammation. In fact, pacritinib, which inhibits IRAK1, demonstrates inhibitory effects against IL-6, TNF, and IL-1β. This is achieved through the swift activation, phosphorylation, and ubiquitination of IRAK1 induced by abundant ssRNA from SARS-CoV-2 and the suppression of HIV-1 GU (HIV-1 Group Undetermined) [71].

## 3. IRAK1 Inhibitors and Degraders

### 3.1. Dual IRAK1/4 Inhibitors

**Figure 2:**
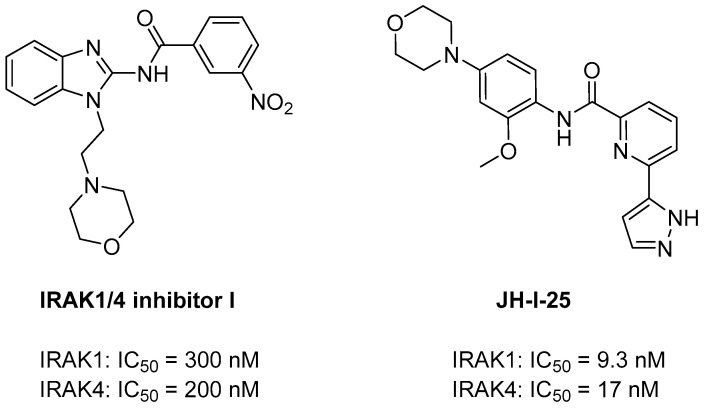
Structures of dual IRAK1/4 inhibitors.

Catalytically active IRAK1 and IRAK4 act as key links in IL-IR signaling and TLR-mediated signaling. They exhibit several structural similarities. The residues that form the ATP-binding pocket exhibit 90% identity between IRAK1 and IRAK4, although IRAK family members show a general sequence identity of approximately 30% [80]. Therefore, many IRAK4 inhibitors exhibit similar potencies against IRAK1. In 2006, J. P. Powers et al. synthesized the dual inhibitor IRAK 1/4 Inhibitor I (Figure 2). IRAK 1/4 Inhibitor I is a novel benzimidazole with IC_50_ values of 300 and 200 nM for IRAK1 and -4, respectively. This compound was identified during the screening of a small-molecule library for the discovery of a series of novel acyl-2-aminobenzimidazole inhibitors of IRAK-4 [81]. IRAK 1/4 Inhibitor I exerts therapeutic potency against HCC and head and neck squamous cell carcinoma (HNSCC) tumors [82,83]. In 2008, G. M. Buckley et al. developed another dual inhibitor, JH-1-25, for the synthesis of IRAK4 inhibitors based on their amide structures (Figure 2). It targets both IRAK1 and IRAK4, with measured IC_50_ values of 9.3 nM and 17.0 nM, respectively, which was determined using the Invitrogen Lantha assay for IRAK1 and the Z′-LYTE assay for IRAK4 [84]. JH-I-25 exhibited binding affinity for both IRAK1 and IRAK4, facilitated by the conserved ATP pocket in IRAK1 and IRAK4. This binding is achieved through a π–π stacking interaction with the gatekeeper residue Y288, along with formation of hydrogen bonds in the hinge region and with the catalytic lysine K239. Additionally, JH-I-25 was acknowledged as the first co-crystal structure with IRAK1 and was recently reported (PDB ID: 6BFN) [80].

### 3.2. Selective IRAK1 Inhibitors

#### 3.2.1. Pacritinib

**Figure 3:**
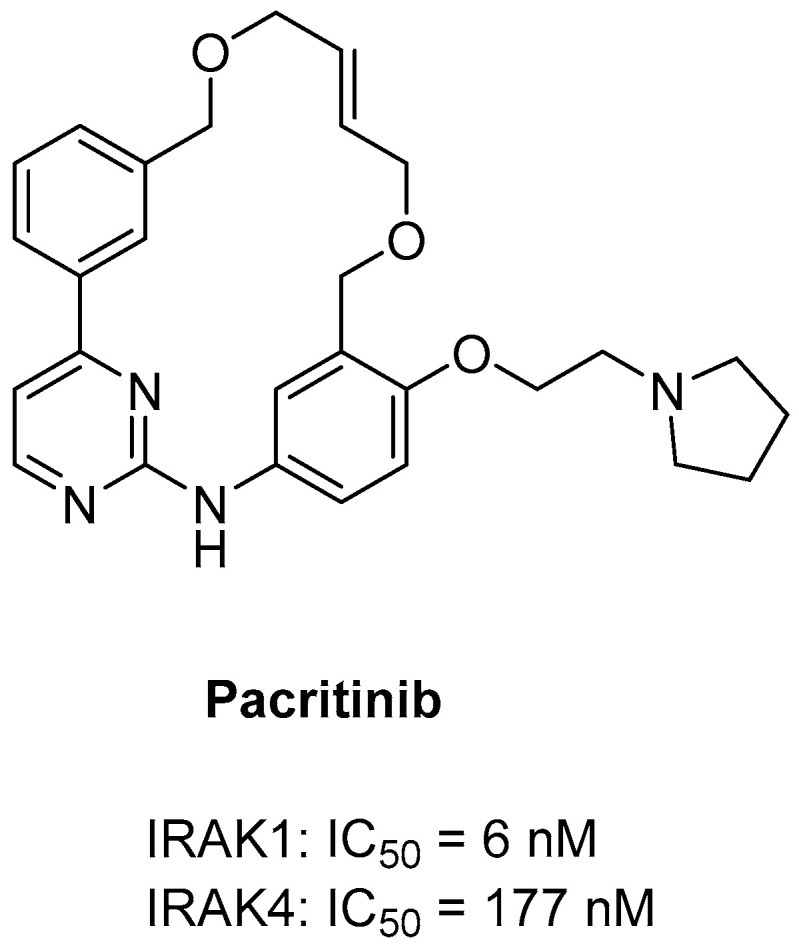
Structure of pacritinib.

Pacritinib is a potent multikinase inhibitor with strong specificity for JAK2/FLT3. It primarily exhibits clinical efficacy against myelofibrosis [80]. Pacritinib was initially approved in the United States in February 2022 to treat adults diagnosed with an intermediate or high risk of primary or secondary myelofibrosis [85]. A recent kinome-wide screening study showed that pacritinib selectively inhibited IRAK1 over IRAK4. Pacritinib inhibited IRAK1 with moderate selectivity against IRAK4 (IC_50_ = 6 nM and 177 nM, respectively), suppressing NF-κB, p38, STAT3, and downstream inflammatory cytokine cascades (Figure 3). Notably, this occurred through a JAK-independent process [86,87]. By selectively inhibiting IRAK1, pacritinib demonstrates efficacy not only in myelofibrosis but also in several other diseases.

Specifically, pacritinib exerts antiproliferative effects in hematologic malignancies such as AML [88]. AML cells exhibit elevated IRAK1 expression, which is a crucial survival signal for AML cells. IRAK1 plays a pivotal role in supporting the viability of leukemic cells, and IRAK1 inhibition could prove advantageous across diverse AML subtypes [70]. Additionally, activated FLT3 kinase has been identified as another molecular target for treating AML, and the concurrent inhibition of JAK2 signaling with FLT3 kinase blockade by pacritinib could enhance its clinical advantages in individuals with AML [88]. Thus, pacritinib is a potent kinase inhibitor with strong specificity for JAK2/FLT3 and IRAK1. It inhibits the growth of AML cells harboring various genetic mutations. This extended effectiveness may result from the ability of pacritinib to target various kinases including IRAK1 [70].

In addition to hematologic malignancies, pacritinib exerts therapeutic effects against breast cancer. Elevated IRAK1 phosphorylation levels are linked to breast cancer recurrence, highlighting the significant role of IRAK1-directed NF-κB signaling in breast cancer metastasis, resistance to chemotherapy, and tumor reappearance. Pacritinib inhibits constitutively activated IRAK1 phosphorylation in breast cancer cells [57,89]. Furthermore, pacritinib may be potentially used as a therapeutic agent for HIV or SARS-CoV-2 by inhibiting IRAK1, which consequently prevents excessive TLR8 signaling. Emerging evidence suggests that abnormal activation of TLR8 and NLRP3 inflammasomes plays a role in both COVID-19 and HIV-1 infections. Therefore, targeting IRAK1, which is a key component of TLR-mediated NLRP3 inflammasome activation, could be a promising therapeutic strategy to alleviate he chronic inflammation associated with SARS-CoV-2 and HIV-1 infections [71].

#### 3.2.2. Rosoxacin

**Figure 4:**
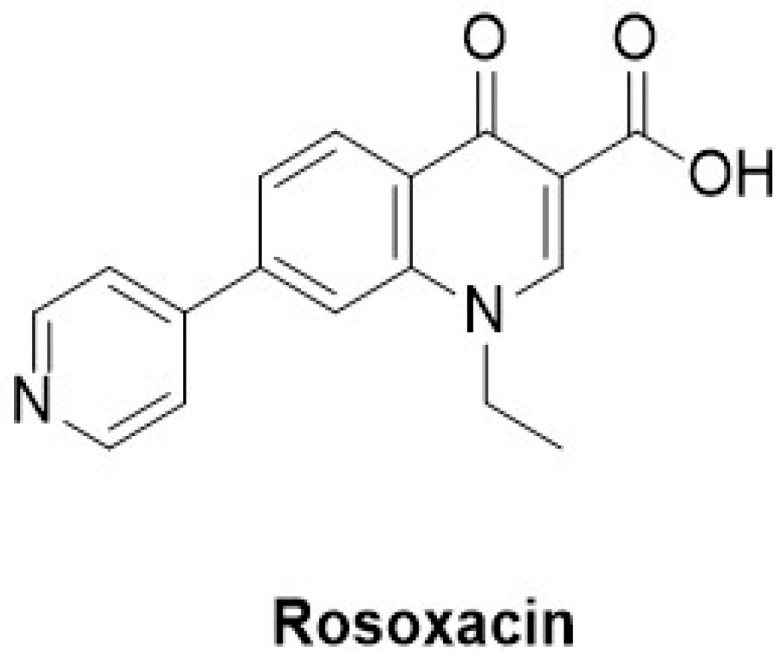
Structure of rosoxacin.

Rosoxacin belongs to the class of first-generation quinolone-derived antibiotics used to treat bacterial infections [90] (Figure 4). However, Hsiao-Chen Huang et al., in 2022, used rosoxacin as a selective inhibitor of IRAK1 and determined its efficacy in treating autoimmune hypophysitis (AH). Chen et al. used a mouse model of experimental autoimmune hypophysitis (EAH) and found an upregulation of IRAK1 in the pituitaries of mice that manifested EAH. They intended to identify IRAK inhibitors by docking 2122 FDA drugs obtained from the Protein Data Bank to the binding site of IRAK1 (PDB ID: 6BFN) using the in-house software GEMDOCK (http://gemdock.life.nctu.edu.tw/dock/ (accessed on 4 May 2024)) [40,91,92]. Among these candidates, rosoxacin emerged as the leading candidate based on the predictions. At 10 μM, rosoxacin demonstrated approximately 50% inhibition of IRAK1 activity, which is significantly higher compared with the <5% inhibition observed for the other tested kinases. Moreover, rosoxacin exhibited approximately 50-fold selectivity for IRAK1 than for IRAK4. Rosoxacin administration during the initial phase (days 0–13) marginally decreased disease severity, whereas treatment during a later phase (days 14–27) demonstrated a notable suppression of EAH. Chen et al. concluded that IRAK1 inhibition by rosoxacin could potentially hinder EAH progression by preventing autoreactive T-cell activation and/or differentiation in the lymph nodes draining the pituitary gland or by impeding T-cell infiltration into the pituitary gland [40].

#### 3.2.3. 1,4-Naphthoquinone

**Figure 5:**
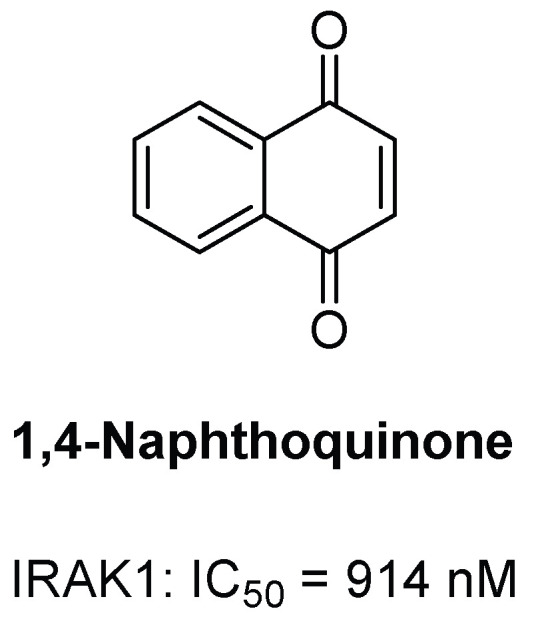
Structure of 1,4-naphthoquinone.

In 2021, Mahmoud et al. screened benzoquinones and naphthoquinone analogues and found 1,4-naphthoquinone to be a selective IRAK1 inhibitor [93] (Figure 5). In an enzyme activity assay, 1,4-naphthoquinone exhibited 91% inhibition at 10 µM against IRAK1, while it inhibited only 10% against IRAK4. Subsequent evaluation of IC_50_ for IRAK1 proved to be 0.914 µM. The findings propose a novel mechanism through which the 1,4-naphthoquinone agent manifests its anti-inflammatory effects, achieved by inhibiting the IRAK1 enzyme and consequently reducing the production of crucial pro-inflammatory cytokines.

### 3.3. Covalent IRAK1 Inhibitor

**Figure 6:**
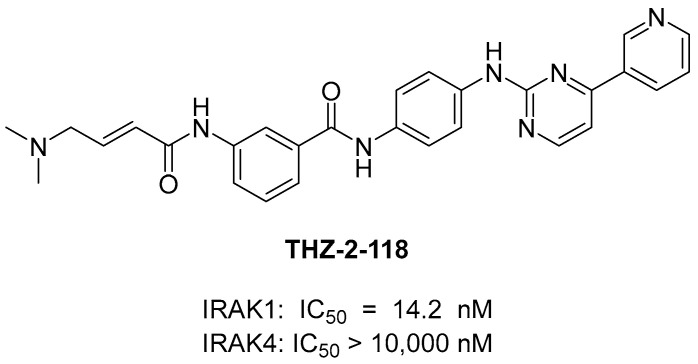
Structure of THZ-2-118.

**Figure 7:**
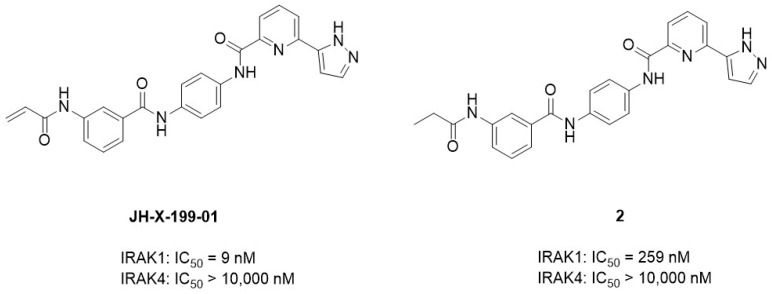
The structures of JH-X-199-01 and its reversible version JH-X-199-01.

In 2020, Hatcher et al. [24] discovered a selective covalent IRAK1 inhibitor JH-X-199-01. IRAK1 is overexpressed in B-cell lymphomas such as Waldenström’s macroglobulinemia (WM) and ABC subtype diffused large B-cell lymphoma (DLBCL) cells [94]. They recognized the significance of IRAK1 inhibition and aimed to develop a selective IRAK1 inhibitor. John et al. used the dual IRAK1/JNK inhibitor “THZ-2-118” as the lead compound. THZ-2-118 exhibited selectivity for IRAK1 over IRAK4, potently inhibiting IRAK1 with an IC_50_ of 14.2 nM. (Figure 6). However, its high potency against JNK1/2/3 kinases should be eliminated before it can be used as an IRAK1 probe. To enhance its selectivity for IRAK1, Hatcher et al. [24] integrated the characteristics of the THZ series with the superior kinome selectivity of the dual IRAK1/4 inhibitor JH-1-25. The resulting compound JH-X-119-01 demonstrated biochemical inhibition of IRAK1, with an apparent IC_50_ of 9 nM. Additionally, IRAK4 inhibition was not observed at concentrations ≤ 10 μM (Figure 7). They performed an LC-MS analysis of labeled IRAK1 and found that JH-X-119-01 formed a covalent bond with the protein. Following digestion and nanoflow LC-MS/MS analysis, they verified that this hybrid compound irreversibly labeled IRAK1 predominantly at C302 (95%) rather than at C307 (5%). They prepared compound **2**, which is a reversible version of JH-X-199-01, to validate the importance of the covalent bonding. The acrylamide warhead in JH-X-199-01 was replaced with a propyl amide. Compound **2** showed a 25-fold loss of potency in IRAK1 inhibition [24] (Figure 6). This demonstrated the significance of the covalent bonds in this compound.

Hatcher et al. [24] tested this compound in a panel of MYD88-expressing mutant ABC DLBCL and lymphoma cells. As MYD88 functions as a signaling adaptor protein in the IRAK-dependent pathway, MYD88 mutations are most frequent in malignancies. JH-X-119-01 showed moderate cell-killing effects in these cells, with an EC_50_ of 12.10 μM in the ABC-DLBCL cell line HBL-1 [24,95,96]. Additionally, as BTK inhibition has been previously shown to synergize with IRAK inhibition, JH-X-119-01 was co-treated with the BTK inhibitor ibrutinib, which showed synergistic tumor cell-killing effects in MYD88-mutated B-cell lymphoma cells [35].

Moreover, JH-X-119-01 exhibited in vivo efficacy against sepsis. Sepsis is a challenging clinical syndrome caused by severe bacterial infection and has a high mortality rate of 30–50%. The primary issue in sepsis is the accompanying systemic inflammatory response syndrome (SIRS), which causes septic shock, multi-organ failure, and death. Bacterial lipopolysaccharide (LPS) from Gram-negative bacteria is a major factor that triggers SIRS [97]. As IRAK1 and IRAK4 play key roles in the LPS-mediated TLR4 pathway, knockout of IRAK1 or IRAK4 in mice reduces LPS-induced oxidative tissue damage and the production of pro-inflammatory cytokines [98]. However, this inhibitory effect on IRAK-4 may lead to a broad and unacceptable impact because IRAK-4 is an upstream activator in multiple signaling pathways [99]. Humans with IRAK4 deficiency exhibit a compromised response to LPS stimulation, making them more susceptible to bacterial infections [100].

Bin Pan et al. [23] tested the impact of a novel selective IRAK1 inhibitor JH-X-119-01 on LPS-induced sepsis in mice and compared the effects of a dual IRAK1/4 inhibitor with those of the IRAK1 selective inhibitor JH-X-119-01. Administering JH-X-119-01 to LPS-challenged mice increased their survival, with higher doses showing enhanced effects. Histological analysis revealed reduced scores for lung injury. Notably, a non-selective IRAK1/4 inhibitor showed similar effects on survival and lung injury in mice with sepsis. However, mice treated with the IRAK1 inhibitor showed elevated white blood cell and CD11b+ myeloid cell counts compared to those treated with the IRAK1/4 inhibitor. Apoptosis analysis in the IRAK1/4 dual inhibitor-treated murine monocyte/macrophage leukemia cell line RAW 264.7 and human acute monocytic leukemia cell line THP-1 showed higher induction of apoptosis compared to when treated with the IRAK1 selective inhibitor JH-X-119-01. This suggests that JH-X-119-01 is less toxic to macrophages than the non-selective IRAK1/4 inhibitors [23].

### 3.4. Selective IRAK1 Degrader

A commonly observed driver mutation in ABC-DLBCL is associated with mutations in the MyD88 gene, and IRAK1 plays a crucial role in ABC-DLBCL with MyD88 mutations [95].

**Figure 8:**
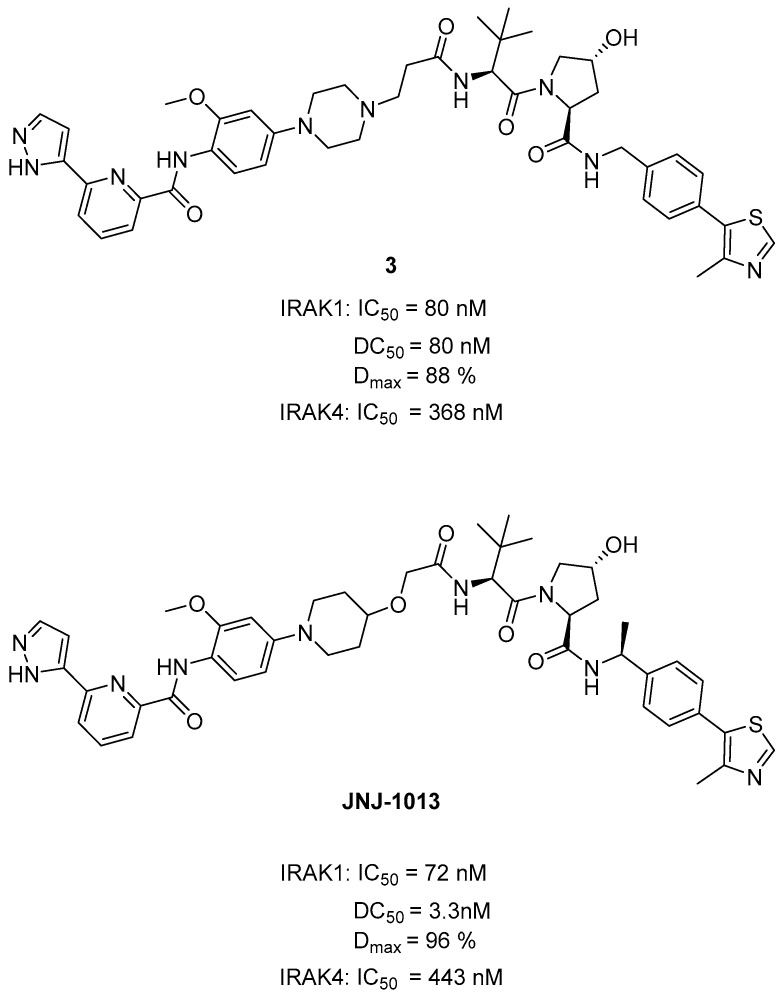
Structures of compound **3** and JNJ-1013.

In 2021, Fu et al. found that tumor cell survival requires the scaffolding function of IRAK1 rather than its kinase activity. They aimed to eliminate the scaffolding function of IRAK1 and developed IRAK1 PROTACs. The dual IRAK1/4 inhibitor JH-1-25 was selected as the starting point [80,84]. Initially, to enable linker conjugation through amine alkylation, the morpholine in the JH-1-25 structure was substituted with piperazine. The von Hippel–Lindau (VHL) ligand VH032 was selected as the E3 ligase ligand. The piperazine group of the IRAK1 warhead was connected to VH032 and an alkyl linker, resulting in compound **3** (Figure 8). By substituting the N-linked piperazine ring in the linker region of compound **3** with an O-linked piperidine and introducing a methyl group in the *S*-configuration within the VHL binder moiety, they formulated the most potent compound JNJ-1013 (Figure 8). JNJ-1013 effectively targeted IRAK1 in terms of degradation, with a DC_50_ of 3.3 nM, and showed enhanced cytotoxicity (IC_50_ = 60 nM) in the ABC-DLBCL cell line HBL1. In comparison, the selective IRAK1 covalent inhibitor JH-X-119-01 showed only moderate cell-killing effects, with an EC_50_ of 12.1 μM in the HBL-1 cell line. This implies that the inhibition of the kinase alone was insufficient to completely interrupt myddosome signaling and adequately restrain the growth of lymphoma cells. Moreover, the capacity of IRAK1 degraders to treat cancers may potentially depend on the IRAK1 scaffolding function [68].

## 4. Conclusions

The IRAK family plays a crucial role in innate immunity by participating in interleukin-1 signaling and TLR-mediated signaling. The dysregulation of these mechanisms has been associated with numerous inflammatory diseases, contributing to the initiation and progression of various cancers. The regulation of IRAK1 could be a promising therapeutic strategy against various inflammation-related diseases and symptoms, including those that are not discussed here. Inflammation is a common factor in many diseases, and IRAK1 plays a crucial role in regulating inflammatory responses. Therefore, the specific regulation of IRAK1 may be a new approach for treating various inflammatory conditions, particularly for diseases such as AML and breast cancer, which have limited effective treatment options.

The selective inhibition of IRAK1 appears to be a promising strategy, especially for diseases such as sepsis and ABC-DLBCL. This approach avoids the broad and unacceptable impact of IRAK4 inhibition, which can lead to toxicity as a side effect. Analyzing selective IRAK1 inhibitors and degraders has revealed promising avenues for therapeutic intervention. Compounds such as pacritinib and JH-X-119-01 exhibit potent effects on hematological malignancies and autoimmune diseases, highlighting the clinical potential of targeting IRAK1. The emergence of IRAK1 degraders such as JNJ-101 provides novel insights into the IRAK1 scaffolding function. Additionally, it presents a unique strategy for disrupting signaling pathways and inducing cytotoxic effects in specific cancer types. In conclusion, IRAK1 is a promising therapeutic target in various diseases. The selective inhibition or degradation of IRAK1 provides an avenue for safer and more effective interventions, especially in the context of cancer therapy. Thus, the evolving landscape of IRAK1-targeted approaches aids in designing innovative treatments and reducing the complications associated with conventional therapies.
